# Current and future methods of probiotic therapy for necrotizing enterocolitis

**DOI:** 10.3389/fped.2023.1120459

**Published:** 2023-03-02

**Authors:** Nitin Sajankila, Samantha Jane Wala, Mecklin Victoria Ragan, Samuel Grant Volpe, Zachary Dumbauld, Nanditha Purayil, Belgacem Mihi, Gail E. Besner

**Affiliations:** Department of Pediatric Surgery, Center for Perinatal Research, Nationwide Children’s Hospital, Columbus, OH, United States

**Keywords:** necrotizing enterocolitis, NEC, probiotic, intestine, gut, microbiome, dysbiosis

## Abstract

Necrotizing enterocolitis (NEC) is a complex intestinal disease that primarily affects premature neonates. Given its significant mortality and morbidity, there is an urgent need to develop improved prophylactic measures against the disease. One potential preventative strategy for NEC is the use of probiotics. Although there has been significant interest for decades in probiotics in neonatal care, no clear guidelines exist regarding which probiotic to use or for which patients, and no FDA-approved products exist on the market for NEC. In addition, there is lack of agreement regarding the benefits of probiotics in neonates, as well as some concerns about the safety and efficacy of available products. We discuss currently available probiotics as well as next-generation probiotics and novel delivery strategies which may offer an avenue to capitalize on the benefits of probiotics, while minimizing the risks. Thus, probiotics may still prove to be an effective prevention strategy for NEC, although further product development and research is needed to support use in the preterm population.

## Introduction

NEC is a severe inflammatory disorder of the premature intestine with complex pathophysiology and limited treatment options (1). One of the earliest reports of the disease was from Babies Hospital in New York City in 1965 (2). Despite several advances in the care of newborns since this time (3, 4), the overall incidence and mortality due to NEC remain high (5, 6). In contrast to respiratory distress syndrome (RDS), another common disease of prematurity, which was radically improved through the introduction of artificial surfactants (7), no such early preventative measure has yet been developed for NEC. In fact, the overall medical care for NEC has remained largely the same since the term was first coined: withholding feeds, antibiotics, and surgery when indicated. Today, NEC is the most common surgical emergency in neonates and the most common cause of gastrointestinal death in this vulnerable patient population. Given the high mortality of NEC, how difficult it is to treat, the significant financial burden it poses on society, and the long-term morbidity in survivors, there is an urgent need to develop novel preventative measures with an aim to eradicate NEC (8).

As NEC typically occurs in the first several weeks of life and is thought partly to be due to an altered gut microbiome (9–11), one potential and promising preventative measure is the prophylactic use of probiotics in susceptible neonates. Probiotics are defined per the World Health Organization (WHO) as live microorganisms such as bacteria that are given in significant enough quantities to provide a specific health benefit (12, 13). While they have been formally studied in the western world since the early 1900s, it was not until the 1950s that they were first trialed in human neonates (14). More than a half-century later, probiotics have failed to gain traction in the USA for the prevention of NEC (15). However, interest in probiotics has increased over time; in 1997, almost no NICUS in the United States were using probiotics (16), but by 2015, that number had increased to 14% (15). Due to concerns regarding safety and efficacy, lack of clear protocolized guidelines for usage, and unavailability of FDA-approved products, neonatologists, pediatric surgeons, and other stakeholders are at present torn on the role of probiotics in preventing NEC. However, an improved mechanistic understanding of probiotic effects on neonatal intestine and immunity, careful selection and dosing of the most efficacious bacterial strains, and advancements in the production and delivery of next-generation probiotics, may warrant future reconsideration of this understandably cautious position. In this review article, we will explore the scientific rationale for the use of probiotics in human neonates, the current state of data in support or against the usage in human neonates, ongoing concerns and barriers to usage, and the future potential of probiotics in the prevention and eradication of NEC.

## Understanding the pathophysiology of NEC and the rationale for prophylactic use of probiotics

The pathophysiology of NEC is known to be complex. This is in part due to early bacterial colonization and an excessive inflammatory response in the context of a premature gut and immune system. Several risk factors have been identified that increase the likelihood of NEC development, including premature birth, very low birth weight, exposure to asphyxia or hypothermia, and enteral feeding (8). This multifactorial pathophysiology underscores how difficult it is to fully prevent NEC with any one single intervention. However, one core component of the disease that may be modifiable, even in the earliest weeks of life, is the altered microbiome characteristic of NEC (17). Understanding the cause and characterizing the extent of this dysbiosis may be key to both understanding NEC and potentially preventing its occurrence.

When neonates are born, they acquire a small library of bacteria from the mother during delivery, from their environment, and from oral feeds, which rapidly expands in both size and diversity. This immature intestinal microbiome is believed to not only influence the immediate health of the neonate but also its life-long health. Most importantly, however, at this initial stage the microbiome is believed to be modifiable, providing a unique opportunity for early intervention (17). The earliest “pioneer” bacteria that seed the intestinal tract during this initial phase include facultative aerobes such as *Escherichia*, *Enterococcus*, and *Streptococcus*, that shift the intestinal luminal environment to an anaerobic one. This shift subsequently allows obligate anaerobes such as *Clostridium*, *Bacteroides*, and *Bifidobacterium* to thrive (18, 19). However, this process can vary tremendously depending on the specific bacteria that neonates are first exposed to, which is influenced by the mode of delivery. Neonates that are delivered vaginally appear to acquire gut flora that resemble their own mother's vaginal microbiome, whereas those delivered by cesarean section develop intestinal microbial communities with similarities to the maternal skin flora (20, 21). In addition to these early colonizers, breast milk feeding expands exposure to *Bacteroides* and *Bifidobacterium*, as well as lactic acid producers: *Lactobacillus* (i.e., *L. acidophilus*), *Limosilactobacillus* (i.e., *L. reuteri*), and *Lacticaseibacillus* (i.e., *L. rhamnosus*). These early gut bacteria are crucial to neonatal health as they are thought to play a role in educating the neonatal immune system and ensuring the evolution of a diverse intestinal microbiome, particularly through the production of beneficial bacterial metabolites (19).

Unfortunately, several factors can disrupt or alter the expected healthy gut colonization, including maternal disease or dysbiosis, cesarean section delivery, absence of breast milk feeding, prematurity, or early antibiotic use (22). Preterm neonates, the population most at risk for NEC, have several additional factors that contribute to dysbiosis, including early exposure to microbes *in utero* (i.e., preterm premature rupture of membranes or intra-amniotic infection), exposure to hospital microbes through prolonged NICU admissions after birth, and expected delays in enteral feeding due to prematurity. Consequently, preterm neonates acquire an abnormal over-representation of pathogenic facultative anaerobes within their intestines, including *Enterobacter*, *Escherichia*, and *Klebsiella*, all belonging to the Gammaproteobacteria class. Additionally, they have decreased proportions of the strict anaerobes that are a hallmark of the healthy developing microbiome, such as *Bifidobacterium* or *Bacteroides* (23).

**Figure 1:**
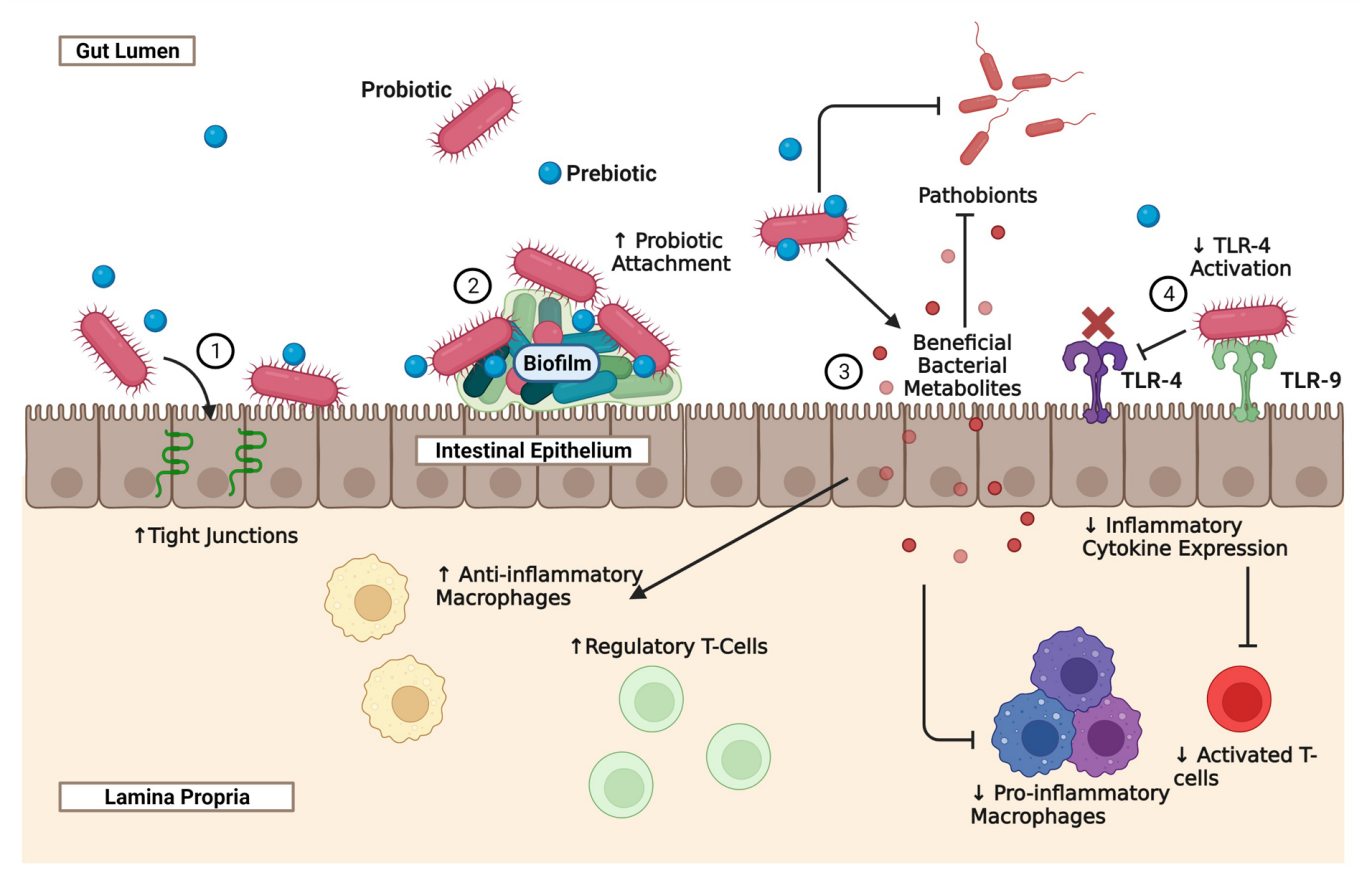
The effect of probiotics and prebiotics on the intestinal epithelium, immune system, and microbiome. Necrotizing Enterocolitis is a complex disease that is in part due to prematurity of the neonatal intestine, prematurity of the developing immune system, and dysbiosis. Probiotics, prebiotics, and synbiotics represent potential novel strategies for modulating all three of the intestine, the immune system, and the microbiome, in order to reduce the incidence and severity of NEC. The mechanisms through which probiotics provide benefits vary depending on the species and strain administered, the adjunct use of prebiotics, the use of novel probiotic delivery systems, and dosing regimens. The schematic illustrates some of the major known effects of probiotics on the developing gut that are relevant to NEC: (1) probiotics can improve gut-barrier function by preserving tight junction proteins such as claudin 4 and occludin. Probiotics also have anti-apoptotic and cytoprotective effects on the neonatal intestine; (2) probiotics that are highly adhesive to the gut intestine produce complex biofilms that improve the attachment and theoretically the efficacy of the probiotic; (3) through direct competition or the production of anti-microbial compounds, probiotics can reduce the presence of pathobionts that contribute to the dysbiosis seen in NEC. Probiotics can also metabolize environmental substrates such as tryptophan to produce beneficial bacterial metabolites that can reduce the presence of pro-inflammatory macrophages and activated T-cells, and increase the populations of anti-inflammatory macrophages and regulatory T-cells; (4) some probiotics are also able to indirectly inhibit the TLR-4 pathway, by interacting with TLR-9. TLR-4 is the receptor for LPS, a microbial cell wall product that is thought to play a role in the pathogenesis of NEC and is commonly used as a stressor in animal models of the disease. By inhibiting TLR-4 activity, there is a reduction in inflammatory cytokines and an increase in regulatory T-cells.

While preterm infants are already noted to have a decreased diversity of intestinal microbes, the insufficiency is further exaggerated in infants that acquire NEC (24). At the same time, the proportion of Gammaproteobacteria in the intestine is further increased, which is predictive of disease development (9–11). Given these findings, there is an opportunity to target therapeutics towards improving the microbial diversity in the gut and reducing the relative abundance of Gammaproteobacteria, in the hope of preventing NEC. One obvious strategy for this is using beneficial bacteria such as *Bacteroides* spp. or *L. reuteri*. Through the production of anti-microbial compounds or direct competition, probiotic bacteria may be able to displace pathogenic bacteria that contribute to the dysbiosis preceding NEC (see Figure 1). For example, *L. reuteri*, in response to various pathogenic-type bacterial strains such as *E. coli*, can generate the antimicrobial compound reuterin, which inhibits bacterial resistance to oxidative stress (25, 26).

In practice, however, it is less clear to what extent this dysbiosis can be transformed into a healthy microbiota and whether this will truly prevent NEC. For example, in one preclinical study that evaluated the ability of a strain of *B. fragilis* to counter *Cronobacter sakazakii*-induced NEC in rodents, pre-treatment with the probiotic slightly improved the loss of microbial diversity and reduced the relative abundance of Proteobacteria. This finding was despite no observable increase in the relative abundance of the probiotic species itself in the gut (27). In another study, administration of *B. infantis* in rodents prevented NEC in a hypoxia-hypothermia model of NEC, but had no impact on dysbiosis, and the probiotic was not detectable in the cecum (28). In contrast, higher dosing of *L. rhamnosus* was not only found to be protective against intestinal injury during experimental NEC, but also resulted in increased microbial diversity. Interestingly, the relative abundance of beneficial bacteria belonging to the phylum Bacteroidetes was also improved compared to lower dosing, underscoring the importance of optimal dosing in characterizing the impact of probiotics on dysbiosis (29). Taken together, these animal studies highlight the variable documented effects of probiotics on the microbiome during NEC, and the difficulty in comparing studies without controlling for differences in specific bacteria used or dosing regimens. Through a careful selection of the most advantageous strains and titration of dosing, the true effects of probiotics on dysbiosis can likely be better assessed in the future.

In addition to dysbiosis, exaggerated inflammation results in significant, patchy, intestinal injury during NEC. Through modulation of the developing immune system and the neonatal intestinal epithelium, prophylactic probiotics may also significantly minimize the intensity of this intestinal inflammation (see Figure 1). Several groups using different probiotic bacteria, including *L. reuteri*, *L. rhamnosus*, and *Bifidobacterium* spp., have shown that prophylactic use of these products can effectively reduce the incidence of NEC, the degree of intestinal injury, and the production of inflammatory cytokines in rodent models of the disease (25, 30–33). However, the mechanisms by which these benefits occur are far less clear, and the effects are likely to be bacterial species or even strain-specific. Several of these probiotic bacteria have been shown to influence gut barrier function, possibly through the regulation of intercellular tight junctions, preventing the translocation of pathogens and resulting sepsis (see Figure 1). For instance, *B. infantis* given to mice prior to initiation of an experimental NEC protocol not only decreased the incidence of NEC, but also reduced the intestinal permeability to the test marker fluorescein isothiocyanate (FITC)-dextran and preserved tight junction proteins such as claudin 4 and occludin (32). Likewise, administration of *L. reuteri* has been shown to decrease intestinal permeability of FITC-dextran during rodent NEC (33). In addition to improving gut barrier function, probiotics can also have anti-apoptotic and cytoprotective effects on the neonatal intestine. For instance, *L. rhamnosus* has been shown to reduce caspase-3 cleavage during experimental NEC and this was associated with an upregulation of pathways involved in epithelial proliferation, migration, growth, and differentiation (34).

Probiotics have also been shown to play a role in modulating the neonatal innate and adaptive immune systems during NEC. For instance, activation of toll-like receptor (TLR) 9 by *L. rhamnosus* DNA has been found to be crucial to its protective abilities against experimental NEC. This is believed to be caused by TLR9 activation resulting in inhibition of TLR4 activation, a receptor that has been implicated in the pathophysiology of NEC and responds to the bacterial cell wall product lipopolysaccharide (LPS) (see Figure 1) (31). The probiotic *L. rhamnosus* can also reduce TLR4 activity during NEC by upregulation of TLR inhibitors such as single immunoglobulin interleukin-1-related receptor (SIGIRR) and A20, and the benefits appear to be dose-dependent (see Figure 1) (29). In addition to enhanced TLR4 activity, diminished regulatory type T cells (Treg), which play a role in modulating the severity of inflammation and promoting tolerance, have also been implicated in the pathophysiology of NEC. Administration of *L. reuteri* (DSM 17938) in a mouse model of the disease was found to reverse this reduction of CD4^+^ Foxp3^+^ Treg cells in the ileum and in mesenteric lymph nodes, which was not seen when *L. acidophilus* DDS was given (see Figure 1) (30). Furthermore, probiotics such as *L. reuteri*, have been shown to beneficially convert substrates such as dietary tryptophan from the environment into bioactive byproducts. Several of these tryptophan breakdown products can bind to a human receptor known as the aryl hydrocarbon receptor and promote an anti-inflammatory state, through reduced TLR-4 signaling in intestinal epithelial cells (35) and reduced inflammatory macrophage infiltration in the intestinal tissue (see Figure 1) (36). Regardless of the mechanisms, the potential for probiotics to beneficially modulate the intestinal epithelium and immune system are additional rationales for the continued development of probiotic therapies against NEC.

## Comparison of current single versus multi-strain probiotics in the prevention of NEC in human neonates

The most studied probiotic bacteria in humans include *Bifidobacterium* spp., *L. reuteri*, or a combination of both (37). These bacteria are normally present in healthy, breastfed, term neonates (38). A study that examined 289 NICUs across the US from 1997 to 2016 found the most commonly administered probiotic products to be *Lactobacillus* (recently recategorized into several genera including *Lactobacillus*, *Limosilactobacillus*, and *Lacticaseibacillus*) formulations followed by Ultimate Flora (*Bifidiobacterium* and *Lactobacillus* spp.), ABC Dophilus (*Bifidobacterium*, *Lactobacillus*, and *Streptococcus* species), and Align (*Bifidobacterium* spp.) (16). Although there is no currently available FDA-approved probiotic, Viswanathan et al. (2016) reported that 14% of NICUs (70/500) in the United States were administering probiotics to very low birthweight (VLBW) infants. Surprisingly, only 4/16 of the probiotics being used in these NICUs were ever evaluated in a randomized controlled trial (RCT) (15). The following sections summarize different RCTs for single and multiple strain probiotic formulations in preterm infants weighing ≤1,500 g [i.e., very low birth weight (VLBW) infants] (see Tables 1, 2).

**Table 1:** Randomized controlled trials studying incidence of NEC using single-strain probiotic formulations in premature neonates.

Probiotic strain	Probiotic dose	Date of publication	Country	Single center vs. multicenter	Number of patients enrolled (probiotic vs. placebo)	Enrollment criteria	Feeding type	Timing of probiotic administration	Incidence of NEC (probiotic vs. placebo)	Incidence of sepsis (probiotic vs. placebo)	References
*L. rhamnosus* GG (Dicoflor™)	6 × 10^9^ CFU daily	2002	Italy	Multicenter	295 vs. 290	GA <33 weeks or birthweight <1,500 g	Both	First enteral feed	1.4% vs. 2.8%, ns	4.7% vs. 4.1%, ns	(39)
*L. rhamnosus* (Dicoflor™)	6 × 10^9^ CFU daily	2006	Italy	Single	39 vs. 41	<1,500 g, >3 days old	Human milk	Third day of life	2.5% vs. 5%, ns	37.5% vs. 42.5%, ns	(40)
*L. reuteri* DSM 17938	1 × 10^8^ CFU daily	2012	Colombia	Multicenter	372 vs. 378	≤2,000 g	Both	Between first 1–2 days of life	3.4% vs. 5.4%, ns (≤1,500 g) 1.5% vs. 2.6%, ns (>1,500 g)	Not reported	(41)
*L. reuteri* DSM 17938	1 × 10^8^ CFU daily	2014	Turkey	Single	200 vs. 200	GA ≤32 weeks, birth weight ≤1,500 g	Both	First enteral feed	4% vs. 5%, ns	6.5% vs. 12.5%, *p *= 0.041	(42)
*L. sporogenes*	3.5 × 10^8^ CFU daily	2011	Turkey	Single	110 vs. 111	GA <33 weeks or birth weight <1,500 g	Breast milk or mixed	First enteral feed	5.5% vs. 9%, ns	26.4% vs. 23.4%, ns	(43)
*B. lactis* BB12	12 × 10^9^ CFU daily	2010	Germany	Single	91 vs. 89	GA <30 weeks	Both	Not reported	2% vs. 4%, ns	Not reported	(44)
*B. breve* BBG-001	10^8^–10^9^ CFU daily	2015	England	Multicenter	650 vs. 660	GA 23-30 weeks	Both	As soon as possible	9% vs. 10%, ns	11% vs. 12%, ns	(45)
*B. breve* OLB6378	2.5 × 10^9^ CFU twice daily	2014	Japan	Multicenter	153 vs. 130	<1,500 g	Both	Within 48 h of birth	0% vs. 0%, ns	8.5% vs. 13.1%, ns	(46)
*S. boulardii* (Reflor™)	5 × 10^9^ CFU daily	2013	Turkey	Single	135 vs. 136	GA ≤32 weeks, birth weight ≤1,500 g	Both	Within 48 h of birth	4.4% vs. 5.1%, ns	34.8% vs. 47.8%, *p *= 0.030 (clinical) 14.9% vs. 15.4%, ns (culture proven)	(47)
*S. boulardii* (Reflor™)	5 × 10^8^ cell/kg twice daily	2013	Turkey	Single	104 vs. 104	GA ≤32 weeks, birth weight ≤1,500	Both	First enteral feed	6.7% vs. 6.7%, ns	24.3% vs. 18.3%, ns	(48)

ns, not significant.

**Table 2:** Randomized controlled trials studying incidence of NEC using multi-strain probiotic formulations in premature neonates.

Probiotic strain	Probiotic dose	Date of publication	Country	Single center vs. multicenter	Number of patients enrolled (probiotic vs. placebo)	Enrollment criteria	Feeding type	Timing of probiotic administration	Incidence of NEC (probiotic vs. placebo)	Incidence of sepsis (probiotic vs. placebo)	References
*B. infantis*, *B. lactis*, and *S. thermophilus* (ABC Dophilus™)	1.0 × 10^9^ CFU daily	2013	Australia, New Zealand	Multicenter	548 vs. 551	GA <32 weeks, weight <1,500 g	Both	When infant was receiving at least 1 ml of milk every 4 h	2% vs. 4.4%, *p *= 0.03	23.5% vs. 26.5%, ns	(49)
*B. infantis*, *S. thermophilus*, and *B. bifidum* (ABC Dophilus™)	1.05 × 10^9^ CFU daily	2005	Israel	Single	72 vs. 73	Birth weight <1,500 g	Both	Recruited on first day of feeds	1% vs. 14%, *p *= 0.013	43% vs. 33%, ns	(38)
*L. acidophilus* and *B. bifidum* (Infloran™)	10^9^ CFU, twice daily	2008	Taiwan	Multicenter	217 vs. 217	GA <34 weeks, birth weight <1,500 g	Breast milk or mixed	Not reported	1.8% vs. 6.5%, *p *= 0.02	19.82% vs. 11.52%, ns	(50)
*L. acidophilus* and *B. bifidum* (Infloran™)	1.0 × 10^9^ CFU of each daily	2014	Thailand	Single	31 vs. 29	Birth weight <1,500 g	Both	First enteral feed	3.2% vs. 3.4%, ns	No sepsis observed in either	(51)
*L. acidophilus B. bifidum* and *B. infantis* (Labinic™)	2 × 10^9^ CFU daily	2022	South Africa	Single	100 vs. 100	GA <37 weeks, birth weight 750–1,500 g	Both	Not reported	0% vs. 5%, ns	Not studied	(52)
*B. longum* and *L. rhamnosus* GG	10^8^ CFU, four times daily	2009	France	Multicenter	45 vs. 49	GA <32 weeks, birth weight <1,500 g	Both	First enteral feed	4.4% vs. 2.0%, ns	33.3% vs. 26.5%, ns	(53)
*B. infantis* (Align™) and *L. rhamnosus* GG (Culturelle™)	5 × 10^8^ CFU of each organism daily	2011	USA	Multicenter	50 vs. 51	Birth weight 501–1,000 g	Not specified	First enteral feed	6% vs. 8%, ns	26% vs. 31%, ns	(54)
*L. acidophilus*, *L. rhamnosus*, *L. casei*, *L. plantarum*, *B. infantis*, *S. thermophilus*	1.0 × 10^9^ CFU/g, 4.4 × 10^8^ CFU/g, 1.0 × 10^9^ CFU/g, 1.76 × 10^8^ CFU/g, 2.76 × 10^7^ CFU/g, 6.6 × 10^5^ CFU/g, respectively, daily	2011	Mexico	Single	75 vs. 75	<1,500 g	Both	First day of enteral feed	8% vs. 16%, ns	56% vs. 58.7%, ns	(55)
*L. acidophilus*, *E. faecium* and *B. infantum*	0.6 × 10^7^ CFU, probiotic strains in ratio of 1.5:1:1.5	2015	Slovenia	Single	40 vs. 40	<1,500 g	Both	First enteral feed	0% vs. 12.5%, *p* = 0.055	40% vs. 72.5%, *p* = 0.006	(56)
*L. acidophilus*, *L. rhamnosus*, *B. longum* and *S. boulardii*	1.25 × 10^9^ CFU daily	2017	India	Single	48 vs. 48	750–1,499 g	Colostrum or donor breast milk	Within 24 h of enteral feed initiation	4.1% vs. 12.5%, ns	Not reported	(57)
*L. casei* and *B. breve* (Yakult LB™)	3.5 × 10^7^ to 3.5 × 10^9^ CFU daily	2011	Brazil	Single	119 vs. 112	750–1,499 g	Breast milk	Second day of life	0% vs. 3.6%, ns	33.6% vs. 37.5%, ns	(58)
*L. acidophilus* and *B. infantis* (Infloran™)	Minimum 1.0 × 10^6^ and 1.0 × 10^6^ of each, respectively, twice daily	2005	Taiwan	Single	180 vs. 187	<1,500 g	Breast milk	Not reported	1.1% vs. 5.3%, *p *= 0.04	12.2% vs. 19.3%, *p *= 0.03	(59)
*B. infantis*, *B. bifidum*, *B. longum* and *L. acidophilus*	2.5 × 10^9^ CFU of each organism, twice daily	2009	India	Single	91 vs. 95	GA <32 weeks, birth weight <1,500 g	Breast milk	Not reported	5.5% vs. 15.8%, *p *= 0.042	14.3% vs. 29.5%, *p *= 0.02	(71)

### Single-strain formulations

Lactic acid producers commonly found in breast milk, including *L. rhamnosus*, *L. reuteri*, and *L. acidophilus* are some of the most common bacteria in probiotic formulations administered in the neonatal population (see Table 1 for comparison of single-strain probiotics in NEC). In 12 NICUs in Italy, 295 VLBW preterm infants were randomized to receive *L. rhamnosus GG* (Dicoflor®; Dicofarm, Rome, Italy), whereas 290 were given placebo. Treatment was given with the first feed, and after at least 7 days of treatment, there was no significant difference in the incidence of NEC. Nevertheless, all patients with NEC in the probiotic group did survive, whereas 25% died in the placebo group (39). Similarly, in a small single-institution RCT with 80 VLBW preterm infants, Dicoflor® reduced gastrointestinal colonization of Candida species. The clinical implications remain unclear as there was no significant difference in the incidence of invasive fungal infections, sepsis, surgical NEC, or death between treatment groups. The lack of significant findings may be attributable to the small study population (40).

The data for other commonly used lactic acid-producing probiotic strains against NEC is similarly mixed. A multi-center, double-blind RCT in Colombia also did not observe a significant decrease in the incidence of NEC between preterm babies who received *L. reuteri* DSM 17938 (Biogaia AB, Stockholm, Sweden) versus placebo. It is important to note that this study was not powered to detect a difference in NEC incidence (41). Likewise, Oncel et al. (2014) investigated the frequency of NEC in a single NICU as a primary outcome in VLBW preterm infants given *L. reuteri* DSM 17938 (Biogaia AB, Stockholm, Sweden) or placebo. After 7 days of treatment, there was no difference in NEC incidence or NEC-related mortality, even after patients were stratified to VLBW or extremely low birth weight (ELBW), defined as neonates weighing <1,000 g. However, there was a significant improvement in sepsis, feeding tolerance, and length of hospitalization in the probiotic arm (42). In contrast, a single-center NICU in Turkey administered *L. sporogenes* (DMG ITALIA SRL, Rome, Italy) to VLBW infants <33 weeks gestational age (probiotic *n* = 110 and control *n* = 111). The incidence of NEC and the incidence of either NEC or death decreased in the probiotic group compared to infants who received the placebo; however, these trends were not statistically significant (*n* = 211) (43). The most recent RCT using *L. reuteri* DSM 17938 demonstrated that this probiotic can modulate the microbiome during the first month of life, improving microbial diversity and reducing the presence of potentially pathogenic bacteria. Although no significant effect on NEC was detected, only 54 neonates were evaluated per group and so this study was underpowered to detect any difference in the occurrence of NEC (61).

The other most studied category of probiotic is *Bifidobacterium* spp. For example, *B. lactis* BB12 was administered to VLBW infants who were <30 weeks gestational age at the Children's Hospital in Ulm, Germany between 2000 and 2003 (probiotic *n* = 91 and placebo *n* = 89). In this study, there was no significant difference in either the incidence of NEC (Bell's stage ≥2) or the incidence of nosocomial infections (primary outcome) between treatment and control groups (44). The largest trial, Probiotics in Preterm Infants (PiPs), investigated the use of *B. breve* BBG-001 (Yakult Honsha Co Ltd, Tokyo, Japan) in 650 babies compared to 660 infants who received placebo across multiple centers in the UK. The group found no protection by the probiotic against sepsis, NEC diagnosis, or death. A limitation of this study was the cross-colonization of the placebo cohort; 49% of infants who received a placebo were colonized with *B. breve* BBG-001 by 36 weeks postmenstrual age (45). A RCT in Japan between 19 NICUs provided *B. bifidum* OLB6378 (Meiji, Tokyo, Japan) (*n* = 153) or placebo (*n* = 130) to VLBW preterm infants within 48 h of life. This study did not identify any difference in NEC incidence, as no infant in either group developed the disease. However, there was significant improvement in feeding tolerance and late-onset sepsis in the probiotic group (46).

Aside from lactic acid producers and *Bifidobacterium*, other beneficial bacteria as probiotics have been studied in RCTs. *Saccharomyces boulardii* (*S. boulardii*) (Reflor®; Biocodex, Beauvois, France), a yeast-based probiotic, was administered to VLBW preterm infants at a single NICU within 48 h of birth. There was no significant difference in NEC (Bell's stage ≥2) or death amongst both groups. There was a significant improvement in feeding tolerance in the probiotic group (47). Another independent RCT also investigating *S. boulardii* (Reflor®; Biocodex, France) did not report a significant difference in the incidence of NEC between the probiotic and placebo group (48).

Overall, the results from current published RCTs on the use of single strain probiotics in preterm infants are not compelling regarding the ability of probiotics to reduce the incidence of NEC. Nonetheless, it is important to note that a number of these studies evaluated NEC only as a secondary outcome and enrolled a small study population. Future, more extensive studies using the most promising strains are warranted to detect any significant changes in the incidence of NEC.

### Multiple-strain formulations

Although the results from RCTs using single-strain formulations have not been significant in decreasing NEC incidence, RCTs in preterm infants using multiple-strain formulations have been more promising (see Table 2 for comparison of multi-strain probiotics in NEC). The ProPerms prospective trial evaluated a combination of *B. infantis*, *B. lactis*, and *Streptococcus thermophilus* (ABC Dophilus; Probiotic Powder for Infants, Solfar, Leonia, New Jersey) in 1,099 VLBW premature infants aged <32 gestational weeks in Australia and New Zealand. Although there was no significant effect on late-onset sepsis, the primary study outcome, the group did demonstrate a significant reduction in NEC in the probiotic group compared to the control (49). In another study, VLBW preterm neonates were randomized to receive ABC Dophilus (Solgar, division of Wyeth Consumer Healthcare, Bergen County, New Jersey), composed of *B. infantis*, *S. thermophilus*, and *B. bifidus*. The treatment group had a lower incidence of NEC (Bell's stage ≥2) and less severe NEC. There was an absolute risk reduction of NEC by 12% in the probiotic cohort (38).

Infloran™, a commonly discussed probiotic formulation composed of *L. acidophilus* and *Bifidobacterium* spp., was retrospectively studied in multiple centers in Germany and showed a significant reduction in the risk of NEC, overall mortality, mortality after NEC, and nosocomial bloodstream infection (62). A multi-center RCT in Taiwan with a total of 434 patients demonstrated similar results using *B. bifidum* and *L. acidophilus* (Infloran, National Collection of Dairy Organisms, Reading, United Kingdom and Laboratorio, Farmaceutico, Mede, Italy) in VLBW preterm infants (50). However, a single-center RCT with VLBW preterm infants using the same formulation did not demonstrate a difference in the incidence of NEC (Bell's stage ≥2). It is worth noting that only 31 infants were randomized to the *B. bifidum* and *L. acidophilus*, and 29 neonates to the placebo group (51). A more recent single-center RCT by Sowden et al. (2022) showed a decrease in the incidence of NEC in VLBW preterm newborns treated with a similar approach using Labinic™ (Bioflortech, Surrey, UK), composed of *L. acidophilus*, *B. bidifum*, and *B. infantis*. Although not statistically significant, zero patients in the probiotic arm had NEC, whereas two in the placebo group were diagnosed with the disease (52).

However, not all studies have found a clear benefit from giving multi-strain probiotic formulations to neonates. Another multi-strain formulation of *B. longum* BB536 and *L. rhamnosus GG* (BB536-LGG; Morinaga Milk Industry Co Ltd., Tokyo, Japan and Valio Ltd.) was studied in VLBW premature infants in two centers in France. There was no difference in the incidence of NEC between the study and the control group. This was partly attributed to a low overall incidence of NEC (53). Another multi-center RCT study showed that *L. rhamnosus GG* (Culturelle; Amerifit, Cromwell, Connecticut) and *B. infantis* (Align; Procter and Gamble, Cincinnati, Ohio) given to ELBW preterm infants did not affect the incidence of NEC or surgery for NEC. Only 101 patients were enrolled in this study, with 51 in the control group and 50 in the probiotic group (54). Several other RCTs have been performed around the world using various formulations of multi-strain probiotics but with low patient enrollments, and have also seen no significant effect on NEC (55–57).

Overall, it appears that the studies using multiple-strain probiotics are more promising than single-strain probiotics; however very few direct comparisons exist at present, making it difficult to recommend one over the other based on individual trial data. Interestingly, a study between single strain *L. acidophilus* and a multispecies probiotic formulation containing *L. acidophilus*, *L. rhamnosus*, *L. casei*, *L. plantroom*, *B. infantis*, and *S. thermophilus*, did not show a significantly different incidence of NEC (63, 64).

### Meta-analysis of single and multiple-strain probiotics in NEC

One of the earliest, high-quality, meta-analyses performed using 7 randomized controlled trial data of preterm neonates that received prophylactic probiotics to prevent NEC, was from 2007 (64). These same data were later updated by the same group in 2010 with the inclusion of 4 additional trials (65). After developing a fixed-effects model using 2,176 preterm neonates with VLBW, they found that the use of probiotics was associated with a lower risk of NEC [RR = 0.35, 95% CI: 0.23–0.55], lower risk of all-cause mortality [RR = 0.42, 95% CI: 0.29–0.62], and an improved time to feed, with a mean difference of 5.03 days saved [−5.03, 95% CI: −5.62 to −4.44]. However, no significant difference was observed regarding impact on sepsis. They concluded that the number needed to treat to prevent 1 case of NEC or 1 death was 25 [95% CI: 17–34] and 20 [95% CI: 14–34], respectively (65).

These findings were validated in another large meta-analysis from 2015 by Lau et al. using 20 RCTs of preterm VLBW infants, in which 12 additional studies were included and 2 from the prior study were not included (66). The most recent Cochrane review from 2020 on this subject including 57 RCTs in total with an expanded study population including very preterm or VLBW infants (*n* = 10,812), added more weight to the emerging importance of probiotics (67). Their analysis revealed that probiotics were associated with a reduction in the risk of NEC [RR = 0.54, 95% CI: 0.45–0.65] and the number needed to treat to prevent one additional case of NEC was 33 [95% CI: 25–50] (67). Through meta-analysis of well-designed RCTs studying the utility of probiotics in preventing NEC in VLBW preterm infants, it is clear that probiotics remain an important strategy for prophylaxis against NEC and deserve continued study.

Interestingly, the work of Lau et al. also highlighted the importance of specific strains and multi-strain formulations in the prevention of NEC (66). Subgroup analyses from this meta-analysis revealed that in particular *Lactobacillus* or mixtures of *Lactobacillus* and *Bifidobacterium*, were most effective in minimizing the risk of NEC (RR = 0.573, 95% CI: 0.354–0.928), in contrast to *Bifidobacterium* alone or *Sacharomyces* alone, which were not significantly effective. Likewise, the multi-strain probiotic recipients had a significantly reduced risk of mortality compared to those that received single-strain formulations (RR = 0.669, 95% CI 0.505–0.886) (66). In fact, more recent meta-analyses have validated the importance of multi-strain formulations of probiotics over single-strain formulations in the prevention of NEC (68, 69). In particular, the 2017 meta-analysis by Chang et al. found *Lactobacillus* species to have a borderline effect against NEC and only multi-strain formulations to be effective in reducing mortality (69). Thus, future studies of probiotics in human neonates should focus on the most effective strains such as *Lactobacillus* species (reclassified into *Lactobacillus*, *Limosilactobacillus*, *Lacticaseibacillus*, among other new and relevant genera) or multi-strain formulations such as *Lactobacillus* and *Bifidobacterium*.

### Confounders and the importance of breast milk in probiotic effects during NEC

There are several factors, regardless of whether single or multi-strain probiotics are used, that complicate analysis and comparison of the RCTs discussed here, including the use of different probiotic formulations and dosing, differences in gestational age of the study groups (degree of prematurity), whether VLBW or ELBW infants were included, and differences in the incidence of NEC. In addition, the use of human breast milk vs. formula to feed the neonate while they are on probiotics may alter the effect of probiotics on NEC (70). For example, probiotic supplementation of *B. breve* and *L. casei* (Yakult LB, São Paulo, Brazil) to human milk in VLBW preterm infants during the first month of life was associated with a reduction in the incidence of NEC (Bell's stage ≥2). In fact, there were only reported cases of NEC in the control cohort (4/112) (58). This was supported by a single-center RCT study, which demonstrated that VLBW infants who received breast milk supplemented with *L. acidophilus* and *B. infantis* (Infloran™; Swiss Serum and Vaccine Institute, Berne, Switzerland) had reduced NEC incidence and rates of NEC or death compared to infants who were fed breast milk alone (59). The importance of breast milk on the function of Infloran™ was again validated in 2015 in another RCT in Europe (60). In contrast, breast milk administration alongside a probiotic mixture of *B. infantis*, *B. bifidum*, *B. longum*, and *L. acidophilus* reduced NEC overall in preterm VLBW infants, but had no difference on Bell's stage ≥2 disease (71). It is possible that concurrent breast milk feeds alongside probiotic administration leads to the improvement of intestinal colonization allowing a greater protection against NEC (72). This is not surprising given the natural role that breast milk has been found to play in preventing NEC. Breast milk provides the developing neonate with valuable maternal IgA (73), immunomodulatory and anti-infective molecules such as lactoferrin (74), beneficial modulation of TLR-4 signaling (75), and specific healthy microbes such as *Lactobacillus* (see section on “*Understanding the pathophysiology of NEC and the rationale for prophylactic use of probiotics”*), packaged alongside the resources that these microbes need to succeed (see section on “*Advances in prebiotics*, *synbiotics*, and *postbiotics”*). Thus, future RCTs should also report the diet of the neonate as an additional variable that might contribute to the bioactivity and success of the probiotic. Overall, more work is clearly needed to identify the most beneficial strain or strains of probiotics to include in future research studies.

## Concerns about sepsis and other major barriers to the use and development of probiotics

The American Academy of Pediatrics (AAP) recently published a statement in November 2021 addressing the use of probiotics in preterm infants. In this statement they decided that at this time they “[do] not support the routine, universal administration of probiotics to preterm infants, particularly those with a birth weight of <1,000 g.” As justification for this conclusion, they cited that most recent modern trials have not demonstrated an apparent reduction in NEC within high-risk infant populations, that there is no pharmaceutical-grade probiotic product currently available in the United States, and that long-term safety remains unknown. However, they did acknowledge that there are conflicting data regarding the use of probiotics in preterm infants for the prevention of NEC. In addition, they encouraged centers choosing to administer probiotics to be selective about their use and to have a thorough discussion of the risks and benefits as a part of a formalized informed consent process (14).

The NEC Society, a non-profit organization dedicated to building a world without NEC *via* research, advocacy, and education, also recently added to this discussion. They acknowledged that further research was required to understand the role of probiotics in the prevention of NEC, to identify which patient populations would benefit most from probiotics, to determine which probiotic strain or strains were preferred, and to confirm the best dose and duration of treatment. However, they did recommend that probiotics be considered as a strategy to help reduce the risks of NEC and death in VLBW infants. Given the lack of clarity, they also recommended that families be better educated about the risks and benefits of probiotic use in NEC, and that clinicians be prepared to explain their NICU's rationale for offering or not offering probiotic administration (76).

This lack of consensus by multiple stakeholders has made it challenging to develop national policies regarding the use of probiotics in neonates. It highlights the essential need for more research on this topic. One of the most piercing concerns from opponents of probiotic use in neonates is the possibility of probiotic-associated sepsis, whether due to contamination or to the possibility of pathogenic behavior by the probiotic bacteria itself. Given that several prior cases of probiotic-associated sepsis or contamination have been documented in the literature, there is good reason to be cautious (77–82). For example, it was reported in 2004 that two pre-term infants in Washington with short bowel syndrome that were given *Lacticaseibacillus rhamnosus* GG to help prevent bacterial overgrowth, developed *L. rhamnosus* GG sepsis (77). The weight of this report was only increased by cases of *L. rhamnosus* GG sepsis after probiotic administration in neonates in Poland in 2014 (78), Italy in 2016 (80), and Taiwan in 2021 (81).

These cases of probiotic sepsis are not exclusively limited to any one species of probiotic bacteria, and have also been seen with currently available commercial formulations. A 2014 report from Switzerland detailed the case of two preterm infants that prophylactically received the probiotic Infloran™, which contains *Bifidobacterium* spp. and *Lactobacillus acidophilus*, to prevent NEC. Both infants unfortunately developed culture-proven *B. longum* bacteremia (82). In 2015, another three cases of *B. longum* bacteremia were reported in preterm infants who received prophylactic Infloran™. Although all three infants had blood cultures positive for *B. longum* either while on Infloran™ or shortly after treatment, two of the three did not require additional antibiotic treatment. The third infant, however, developed NEC, despite treatment with Infloran™, and ultimately required both antibiotics and surgery (79). Although these cases are rare, the existence of these sentinel events is troublesome. Our lack of understanding as to why probiotic-related bacteremia occurs, which subpopulations of premature neonates are at the highest risk, and whether this is even preventable given the loss of intestinal barrier function in NEC, continues to be a significant barrier to the widespread use of probiotics in NICUs.

In addition to hesitancy due to a lack of defined guidelines for the role of probiotics in the treatment of NEC, and the rare but notable cases of probiotic-related sepsis, the absence of government oversight or regulation in this industry is another barrier to usage. At present, there are no FDA-approved probiotics on the market and the precise contents of non-FDA-approved probiotic formulations currently available cannot be guaranteed. Drago et al. conducted a study in 2009 to determine if products available in the USA market were correctly labeled and found that the contents of only 4 of 13 products matched their labels (83). A similar study by Toscano et al. in 2011 investigating products on the Italian and European market found that out of 24 products, 10 did not contain the expected amount of bacteria listed on the label and 4 did not contain any of the species included on the label (84). As recently as 2016, Lewis et al. aimed to validate the identity of *Bifidobacterium* species and subspecies in 16 different commercial products, of which only one probiotic perfectly matched its label (85). Beyond the discordance between product labels and their contents, there have been several probiotic recalls due to contamination (86–88). A widely known incident of probiotic-associated sepsis due to contamination was the death of a VLBW preterm infant in Connecticut, who unfortunately succumbed to gastrointestinal mucormycosis after receiving the probiotic ABC Dophilus Powder that was contaminated with *Rhizopus oryzae* (89).

These uncertainties and discrepancies demonstrate the importance of good manufacturing practice (GMP)-grade probiotic preparation for human administration as an important next step in developing probiotic drugs for NEC. However, given the exorbitant cost of producing a GMP-grade drug formulation, and the enormous effort required to test that drug and get it approved by the FDA, this is a significant hurdle. As our target population is newborns, the cost may be doubled as the FDA requires initial Phase 1 studies in adults prior to beginning Phase 1 studies in newborns (90). Funding this extensive effort is difficult without the support of pharmaceutical companies. Unfortunately, NEC is an orphan disease affecting less than 200,000 infants nationwide (91). As such, there is not a great incentive for pharmaceutical companies, hospitals, and government agencies to support new research and the development of novel therapeutics to treat NEC, compared to therapeutics for more prevalent diseases (90). Despite these clear difficulties in producing a probiotic drug for NEC, several competing groups are working at present to test GMP-grade probiotics in the clinical setting, in order to gain full FDA approval. One such GMP-grade probiotic drug known as IBP-9414 (*L. reuteri*), developed by Infant Bacterial Therapeutics AB (IBT), is currently being studied in an ongoing, registered, phase 3 RCT known as the “Connection Trial” (NCT03978000). This study is presently in the recruiting phase and is slated to be complete by the end of 2023. In addition to uniquely being one of the few studies using GMP-grade products in an RCT, this study is also intentionally being powered to see an effect for NEC (92). If this GMP-grade product achieves full FDA-approval, this could change the landscape for the use of probiotics in NICUs, as it may be more universally accepted amongst neonatologists as a therapeutic option against NEC. Of note, IBP-9414 at present has received orphan drug status for the prevention of retinopathy of prematurity, but not for NEC (93). Preliminary data from this study was limited to establishing definitions for sustained feeding tolerance, a primary outcome for their trial, and researchers have not yet commented on the efficacy of their probiotic against NEC as they remain blinded. However, we do know that their overall incidence of NEC at this time, regardless of allocation to probiotic or control group, is 6% (*n* = 13/216) (94).

## Next-generation probiotics in the prevention of NEC

### Advances in prebiotics, synbiotics, and postbiotics

**Figure 2:**
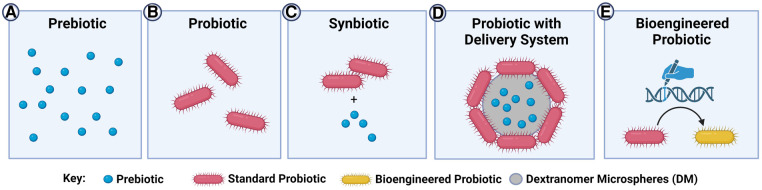
Current and next-generation probiotic-related therapies in the prevention of NEC. (**A**) Prebiotics are substrates that bacteria can utilize to confer a health benefit to the host. Since no bacteria are administered, this strategy eliminates concerns about probiotic sepsis. However, there are limited pathways being targeted compared to the complex interactions resulting from the use of whole bacteria; (**B**) probiotics are live bacterial species that confer a health benefit to the host. These products have a much broader range of targets/effects than simple prebiotics, but there is a theoretical risk of probiotic sepsis; (**C**) synbiotics are probiotics that are co-administered with beneficial prebiotics. The prebiotics can enhance the effect of the probiotic, however the prebiotic may not exclusively be used by the probiotic itself and could be utilized by other intestinal bacteria; (**D**) probiotics can be administered using novel delivery systems such as dextranomer microspheres (DM), which can be pre-loaded with prebiotics. These delivery systems can promote the formation of a biofilm, leading to increased attachment of the probiotic to the intestinal mucosa. Administration in the biofilm state improves survival of the probiotic against the harsh gastric and intestinal environment. The prebiotic and probiotic are co-localized to ensure maximal use of the prebiotic by the adherent probiotics, with no-off target effects of the prebiotic on other microbes; (**E**) bioengineered probiotics are theoretical or emerging probiotics in which specific pathways are enhanced or altered through bioengineering strategies. This can theoretically reduce safety concerns by eliminating pathogenicity and improve efficacy by selecting beneficial phenotypes. However, significant regulatory hurdles for the development and testing of bioengineered probiotics exist at present.

In addition to probiotics, prebiotics, synbiotics and postbiotics have emerged as potential prophylactic strategies against NEC (see Figure 2). A prebiotic is defined as a “substrate that is selectively utilized by host microorganisms conferring a health benefit” (95, 96). Breast milk contains prebiotics known as human milk oligosaccharides (HMOs), with HMO 2’-fucosyllactose (2’FL) being the most predominant (97). HMOs are selectively consumed by *Bifidobacterium* species, which colonize the gut in healthy breastfed infants (98). In an experimental rat model of NEC, HMOs or 2’FL alone were shown to reduce pathology compared to formula-fed only animals (99). Another important component of breast milk, particularly colostrum, is the iron-binding glycoprotein lactoferrin, which can promote the growth of *L. acidophilus* and *Bifidobacterium* species (100). A Cochrane review showed that lactoferrin decreased the incidence of NEC (Bell's stage ≥2) in pre-term infants when added to enteral feeds with or without probiotics (74). Thus, prebiotics remain a promising avenue in the treatment of NEC given their beneficial effects on commensal bacteria. If the right combination of prebiotics were discovered to help assure healthy maturation of the microbiome, it is possible that probiotics might not be needed at all; thus, eliminating the risk of probiotic-related sepsis and contamination.

Synbiotics, on the other hand, are a combination of prebiotic and probiotic products, in which the presence of the prebiotic benefits the growth of both the probiotic bacteria and commensal host flora (101). While this is a promising concept, further evaluation is necessary as the available data on their beneficial role and their innocuity are very limited. A group in Turkey performed a RCT where VLBW infants ≤32 gestational weeks received oral *Lactobacillus* species, *B. lactis*, oligosaccharides, and bovine lactoferrin with feeds. There was no difference between the treatment and control groups in terms of NEC severity, incidence, or death (101). On the contrary, a multi-center, international RCT revealed that bovine lactoferrin alone or in combination with *L. rhamnosus GG* was associated with a significantly reduced incidence of NEC compared to placebo (102). Another RCT found that enteral administration of multi-strain probiotics consisting of *L. rhamnosus*, *L. casei*, *L. plantorum*, and *B. animalis* (NBL probiotic®) alongside fructooligosaccharides and galactooligosaccharides to VLBW preterm neonates resulted in significantly decreased mortality and NEC incidence compared to placebo (103). Careful selection of prebiotic and probiotic combinations is important in the development of synbiotics to ensure long-lasting beneficial effects. For example, in an interim evaluation of an ongoing RCT, the enteral administration of *L. reuteri* in conjunction with *ω*-3 fatty acid treatment prenatally to the mother and then postnatally in the neonate resulted in synergistic epigenetic changes in allergy and immune-related pathways in T-helper cells (104). Thus, synbiotics are a clear new frontier for optimizing probiotic-based interventions for NEC.

Finally, a postbiotic is a bioactive metabolite with beneficial properties produced by a microorganism and used as a direct therapeutic in place of the microorganism (105, 106). For instance, Meng et al. (2020) identified anti-inflammatory indole-3-lactic acid (ILA) as a beneficial breakdown product of tryptophan produced by *B. infantis*. The addition of this postbiotic to enterocytes originating from a NEC patient *in vitro*, prior to addition of interleukin-1β (IL-1β) stress, resulted in reduced IL-8 secretion by the cells (107). Overall, there are fewer studies investigating the effect of postbiotics in NEC compared to prebiotics and synbiotics. However, this line of research will undoubtedly advance the field of probiotics overall, as it will allow for careful selection of strains based on their metabolic products. As we develop a more refined understanding of the optimal substrates and environment required by specific probiotics, we may be able to ensure the success of probiotics and even amplify their effects against NEC.

### Developing novel delivery systems

Probiotics administered enterally face several inherent challenges before successfully colonizing the intestine, including exposure to gastric acids, turbulent intraluminal fluid forces, and competition with other microbes and the host immune response (108). One mechanism that some bacteria naturally employ to survive these harsh conditions, and to successfully attach to the intestinal wall, is the production of biofilms. Biofilms are an extracellular matrix composed of oligosaccharides, proteins, lipids, and DNA, produced by communities of bacteria to enhance their adherence to surfaces such as the intestinal wall (109). Interestingly, biofilms may also play a role in the ability of some probiotics to attenuate intestinal inflammation. In adult mice, highly adhesive strains of *L. reuteri* have been shown to elicit a greater anti-inflammatory IL-10 response after LPS stress, compared to less adhesive strains (110). While several authors have tested strains of *L. reuteri* that happen to be biofilm-producing or highly adhesive, such as DSM 20016, this is not typically a variable that has been prioritized for probiotic selection in humans. However, enhancing biofilm production by *L. reuteri* DSM 20016 may improve the overall efficacy of the probiotic against human NEC by improving intestinal colonization.

One novel approach to capitalize on the inherent adhesiveness of *L. reuteri* DSM 20016 is by growing these bacteria on the surface of dextranomer microspheres such as Sephadex™ (DM), as a vehicle for delivery of the probiotics (see Figure 2) (111). When *L. reuteri* DSM 20016 (*Lr*) is grown on DM (*Lr*-DM) there is enhanced biofilm production (112). In a rat model of the disease, we have shown that a single dose of *Lr*-DM (i.e., *Lr* administered in its biofilm state) administered after birth significantly protects the intestines against NEC (111). While most dosing strategies of probiotics in human NEC require daily usage, this delivery system could radically minimize the exposure of a premature neonate to probiotic bacteria, reducing the risk of probiotic-related sepsis. Also, DM can be loaded with beneficial substances such as maltose (*Lr*-DM-maltose) to further increase biofilm production, and we have shown that this further enhances the ability of the probiotic to protect the intestines against NEC in rats (33). We have now tested *Lr*-DM-maltose in a piglet model of NEC, and have confirmed these promising pre-clinical findings (unpublished observations). Unlike typical synbiotic strategies, where the probiotic and its substrate are fed separately, this delivery system allows for co-localization of the substrate to the microenvironment of the attached bacteria, avoiding any off-target effects of the prebiotic on potentially pathogenic organisms. Through the targeted selection of beneficial strains (i.e., *L. reuteri* DSM 20016) administered using novel delivery systems, it is possible that probiotics can be more safely and effectively delivered to human neonates in a prophylactic fashion.

### Generating designer probiotics

Although not yet studied in the context of NEC, one next-generation approach to fine-tune probiotics to better address diseases while minimizing off-target effects is through genetic engineering. By editing specific disease-related genes, including those involved in inflammation, infection, or metabolism-related pathways, it may be possible to create enhanced probiotic strains, loosely known as designer probiotics, that can better address the diseases they are being developed for (see Figure 2). While there are significant ethical and safety issues with generating new bacterial strains and testing them in humans, the early efforts in this arena are encouraging and are likely to continue to evolve (113). Several examples of designer probiotics exist at present in the pre-clinical arena, targeting a diverse range of inflammatory and non-inflammatory diseases. For instance, the *L. plantarum* NC8 strain was modified to produce angiotensin-converting enzyme inhibitory peptides to successfully combat hypertension in rats (114) and *B. longum* was engineered to secrete fully functional glucagon-like peptide-1 to improve pancreatic function in type 2 diabetes mellitus (115).

With regards to addressing inflammatory and infectious disorders of the gut that might be relevant to NEC, *L. lactis* was modified to serve as a prophylactic vaccine against *C. difficile*, through the expression of non-toxic fragments of *C. difficile* cytotoxins. It was shown in an *in vivo* mouse model that this vaccination strategy improved survival and resulted in increased IgG and IgA titers (116). Another example of a bioengineered probiotic that might be relevant to NEC was the recent modifications of *E. coli* Nissle 1917, a harmless gram-negative bacterium, that was developed to combat *C. difficile* colonization (117). Given that conjugated bile acids have been found to play a role in *C. difficile* colonization, *E. coli* Nissle 1917 was bioengineered to deconjugate intestinal bile acids. Furthermore, it was modified to perform this deconjugation task only when dysbiosis was observed, through the detection of subtle changes in intraluminal sialic acid concentration, a reliable biomarker for dysbiosis (117). When testing this remarkable dysbiosis-sensing probiotic against *C. difficile in vitro*, it was found that the pathogen's germination and growth were significantly inhibited, and its toxicity was reduced. Most importantly, administration of this probiotic reduced histologic injury after *C. difficile* infection in mice (117). Another relevant approach that has been employed to reduce pathogen toxins in the intestine is the development of probiotics that express toxin receptor mimics to neutralize the toxin and minimize its binding to host toxin receptors (118).

As we develop a more rigorous understanding of NEC pathogenesis, it may be possible to create similar engineered probiotics that respond to early NEC-related changes, with targeted responses to neutralize pathogens or toxins and strengthen host defenses. Recently, it was shown that NEC may be associated with a reduction in IL-22 signaling and that recombinant IL-22 therapy during NEC could significantly reduce the severity of experimental NEC in mice (119). It will be interesting to study how probiotics engineered to deliver IL-22 or other disease-mitigating products might perform against NEC, a strategy that was very recently utilized with a modified IL-22 producing *L. reuteri* to protect against intestinal radiation in mice (120). While these “designer probiotics” are exciting alternatives as they might radically improve the efficacy of probiotics against NEC, it is important not to minimize the sheer volume of regulatory hurdles and preclinical work that would be required prior to such products being tested in neonates.

## Conclusion

Despite decades of research on the use of probiotics in humans, the role of probiotics in preventing NEC remains controversial and unclear. Differences in dosing strategies, use of single versus multi-strain formulations, and co-administration of prebiotics or breast milk, have complicated comparisons and interpretations of previous work. However, the abundance of data available has helped to identify several specific strains of probiotic that merit further testing based on their anti-inflammatory, anti-microbial, metabolic, or highly adhesive properties. Current ongoing work in the field of probiotics has sought to amplify the effects of these strains and minimize concerns about safety, through the generation of next-generation synbiotics, delivery systems, and designer probiotics. Through careful strain selection and optimization of dosing strategies and effects, it is quite possible to use probiotics to effectively prevent NEC. FDA approval, GMP-grade production, and evidence-based guidelines are likely to significantly increase the routine use of probiotics in neonates in the future.

## References

[1] de Plaen IG. Inflammatory signaling in necrotizing enterocolitis. Clin Perinatol. (2013) 40(1):109. 10.1016/J.CLP.2012.12.008 23415267 PMC3579498

[2] Mizrahi A Barlow O Berdon W Blanc WA Silverman WA. Necrotizing enterocolitis in premature infants. J Pediatr. (1965) 66(4):697–706. 10.1016/S0022-3476(65)80003-8 14271359

[3] Lussky RC Cifuentes RF Siddappa AM. A history of neonatal medicine—past accomplishments, lessons learned, and future challenges. Part 1—the first century. J Pediatr Pharmacol Ther. (2005) 10(2):76. 10.5863/1551-6776-10.2.76 23118629 PMC3468063

[4] Neu J Modi N Caplan M. Necrotizing enterocolitis comes in different forms: historical perspectives and defining the disease. Semin Fetal Neonatal Med. (2018) 23(6):370–3. 10.1016/J.SINY.2018.07.004 30100524

[5] Stoll BJ. Epidemiology of necrotizing enterocolitis. Clin Perinatol. (1994) 21(2):205. 10.1016/S0095-5108(18)30341-5 8070222 PMC7133385

[6] Guthrie SO Gordon PV Thomas V Thorp JA Peabody J Clark RH. Necrotizing enterocolitis among neonates in the United States. J Perinatol. (2003) 23(4):278–85. 10.1038/SJ.JP.7210892 12774133

[7] Schwartz RM Luby AM Scanlon JW Kellogg RJ. Effect of surfactant on morbidity, mortality, and resource use in newborn infants weighing 500 to 1500 g. N Engl J Med. (1994) 330(21):1476–80. 10.1056/NEJM199405263302102 8164699

[8] Neu J Walker WA. Necrotizing enterocolitis. N Engl J Med. (2011) 364(3):255–64. 10.1056/NEJMRA1005408 21247316 PMC3628622

[9] Warner BB Deych E Zhou Y Hall-Moore C Weinstock GM Sodergren E Gut bacteria dysbiosis and necrotising enterocolitis in very low birthweight infants: a prospective case-control study. Lancet. (2016) 387(10031):1928–36. 10.1016/S0140-6736(16)00081-7 26969089 PMC5553277

[10] Mai V Young CM Ukhanova M Wang X Sun Y Casella G Fecal microbiota in premature infants prior to necrotizing enterocolitis. PLoS One. (2011) 6(6):e20647. 10.1371/JOURNAL.PONE.0020647 21674011 PMC3108958

[11] Morrow AL Lagomarcino AJ Schibler KR Taft DH Yu Z Wang B Early microbial and metabolomic signatures predict later onset of necrotizing enterocolitis in preterm infants. Microbiome. (2013) 1(1):1–16. 10.1186/2049-2618-1-13 24450576 PMC3971624

[12] Hill C Guarner F Reid G Gibson GR Merenstein DJ Pot B Expert consensus document. The international scientific association for probiotics and prebiotics consensus statement on the scope and appropriate use of the term probiotic. Nat Rev Gastroenterol Hepatol. (2014) 11(8):506–14. 10.1038/NRGASTRO.2014.66 24912386

[13] Food and Agricultural Organization of the United Nations and World Health Organization. Joint FAO/WHO working group report on drafting guidelines for the evaluation of probiotics in food. Food and Agricultural Organization of the United Nations (2002). Available at: https://www.who.int/foodsafety/fs_management/en/probiotic_guidelines.pdf (Accessed January 15, 2023).

[14] Poindexter B Cummings J Hand I Adams-Chapman I Aucott SW Puopolo KM Use of probiotics in preterm infants. Pediatrics. (2021) 147(6):1–7. 10.1542/PEDS.2021-051485/180282 34031231

[15] Viswanathan S Lau C Akbari H Hoyen C Walsh MC. Survey and evidence based review of probiotics used in very low birth weight preterm infants within the United States. J Perinatol. (2016) 36(12):1106–11. 10.1038/JP.2016.144 27583387

[16] Gray KD Messina JA Cortina C Owens T Fowler M Foster M Probiotic use and safety in the neonatal intensive care unit: a matched cohort study. J Pediatr. (2020) 222:59–64.e1. 10.1016/J.JPEDS.2020.03.051 32418818 PMC7321859

[17] Oliphant K Claud EC. Early probiotics shape microbiota. Nat Microbiol. (2022) 7(10):1506–7. 10.1038/S41564-022-01230-9 36163499

[18] Karlsson CLJ Molin G Cilio CM Ahrné S. The pioneer gut microbiota in human neonates vaginally born at term-a pilot study. Pediatr Res. (2011) 70(3):282–6. 10.1203/PDR.0B013E318225F765 21629156

[19] Houghteling PD Walker WA. Why is initial bacterial colonization of the intestine important to the infant’s and child’s health? J Pediatr Gastroenterol Nutr. (2015) 60(3):294. 10.1097/MPG.0000000000000597 25313849 PMC4340742

[20] Dominguez-Bello MG Costello EK Contreras M Magris M Hidalgo G Fierer N Delivery mode shapes the acquisition and structure of the initial microbiota across multiple body habitats in newborns. Proc Natl Acad Sci U S A. (2010) 107(26):11971–5. 10.1073/PNAS.1002601107 20566857 PMC2900693

[21] Rutayisire E Huang K Liu Y Tao F. The mode of delivery affects the diversity and colonization pattern of the gut microbiota during the first year of infants’ life: a systematic review. BMC Gastroenterol. (2016) 16:1–12. 10.1186/S12876-016-0498-0 27475754 PMC4967522

[22] Walker WA. The importance of appropriate initial bacterial colonization of the intestine in newborn, child and adult health. Pediatr Res. (2017) 82(3):387. 10.1038/PR.2017.111 28426649 PMC5570628

[23] Cuna A Morowitz MJ Ahmed I Umar S Sampath V. Microbiome and host interactions: dynamics of the preterm gut microbiome in health and disease. Am J Physiol Gastrointest Liver Physiol. (2021) 320(4):G411. 10.1152/AJPGI.00399.2020 33439103 PMC8238167

[24] Wang Y Hoenig JD Malin KJ Qamar S Petrof EO Sun J 16S rRNA gene-based analysis of fecal microbiota from preterm infants with and without necrotizing enterocolitis. ISME J. (2009) 3(8):944–54. 10.1038/ISMEJ.2009.37 19369970 PMC2713796

[25] Shelby RD Mar P Janzow GE Mashburn-Warren L Tengberg N Navarro JB Antibacterial and anti-inflammatory effects of *Lactobacillus reuteri* in its biofilm state contribute to its beneficial effects in a rat model of experimental necrotizing enterocolitis. J Pediatr Surg. (2022) 57(7):1382–90. 10.1016/J.JPEDSURG.2021.09.001 34657737

[26] Schaefer L Auchtung TA Hermans KE Whitehead D Borhan B Britton RA. The antimicrobial compound reuterin (3-hydroxypropionaldehyde) induces oxidative stress via interaction with thiol groups. Microbiology. (2010) 156(Pt 6):1589. 10.1099/MIC.0.035642-0 20150236 PMC7336520

[27] Fan H Chen Z Lin R Liu Y Wu X Puthiyakunnon S Bacteroides fragilis strain ZY-312 defense against Cronobacter sakazakii-induced necrotizing enterocolitis in vitro and in a neonatal rat model. mSystems. (2019) 4(4):1–16. 10.1128/MSYSTEMS.00305-19/ASSET/D2F89B7A-5164-4FD2-B038-F8BDC8322268/ASSETS/GRAPHIC/MSYSTEMS.00305-19-F0006.JPEG PMC6687943 31387931

[28] Underwood MA Arriola J Gerber CW Kaveti A Kalanetra KM Kananurak A Bifidobacterium longum subsp. infantis in experimental necrotizing enterocolitis: alterations in inflammation, innate immune response, and the microbiota. Pediatr Res. (2014) 76(4):326–33. 10.1038/PR.2014.102 25000347 PMC4167942

[29] Cuna A Yu W Menden HL Feng L Srinivasan P Chavez-Bueno S NEC-like intestinal injury is ameliorated by Lactobacillus rhamnosus GG in parallel with SIGIRR and A20 induction in neonatal mice. Pediatr Res. (2020) 88(4):546–55. 10.1038/S41390-020-0797-6 32053825 PMC8213439

[30] Liu Y Fatheree NY Dingle BM Tran DQ Rhoads JM. Lactobacillus reuteri DSM 17938 changes the frequency of Foxp3+ regulatory T cells in the intestine and mesenteric lymph node in experimental necrotizing enterocolitis. PLoS One. (2013) 8(2):e56547. 10.1371/JOURNAL.PONE.0056547 23437165 PMC3577854

[31] Good M Sodhi CP Ozolek JA Buck RH Goehring KC Thomas DL Lactobacillus rhamnosus HN001 decreases the severity of necrotizing enterocolitis in neonatal mice and preterm piglets: evidence in mice for a role of TLR9. Am J Physiol Gastrointest Liver Physiol. (2014) 306(11):1021–32. 10.1152/AJPGI.00452.2013 PMC4042115 24742987

[32] Bergmann KR Liu SXL Tian R Kushnir A Turner JR Li HL Bifidobacteria stabilize claudins at tight junctions and prevent intestinal barrier dysfunction in mouse necrotizing enterocolitis. Am J Pathol. (2013) 182(5):1595–606. 10.1016/J.AJPATH.2013.01.013 23470164 PMC3644725

[33] Olson JK Navarro JB Allen JM McCulloh CJ Mashburn-Warren L Wang Y An enhanced Lactobacillus reuteri biofilm formulation that increases protection against experimental necrotizing enterocolitis. Am J Physiol Gastrointest Liver Physiol. (2018) 315(3):G408–19. 10.1152/ajpgi.00078.2018 29848024 PMC6415713

[34] Lin PW Nasr TR Berardinelli AJ Kumar A Neish AS. The probiotic Lactobacillus GG may augment intestinal host defense by regulating apoptosis and promoting cytoprotective responses in the developing murine gut. Pediatr Res. (2008) 64(5):511–6. 10.1203/PDR.0B013E3181827C0F 18552706 PMC2694849

[35] Lu P Yamaguchi Y Fulton WB Wang S Zhou Q Jia H Maternal aryl hydrocarbon receptor activation protects newborns against necrotizing enterocolitis. Nat Commun. (2021) 12(1):1–14. 10.1038/s41467-021-21356-4 33589625 PMC7884836

[36] Nolan LS Mihi B Agrawal P Gong Q Rimer JM Bidani SS Indole-3-carbinol-dependent aryl hydrocarbon receptor signaling attenuates the inflammatory response in neonatal necrotizing enterocolitis. Immunohorizons. (2021) 5:193–209. 10.4049/IMMUNOHORIZONS.2100018 33906960 PMC8173979

[37] Patel RM Underwood MA. Probiotics and necrotizing enterocolitis. Semin Pediatr Surg. (2018) 27(1):39–46. 10.1053/J.SEMPEDSURG.2017.11.008 29275816 PMC5844696

[38] Bin-Nun A Bromiker R Wilschanski M Kaplan M Rudensky B Caplan M Oral probiotics prevent necrotizing enterocolitis in very low birth weight neonates. J Pediatr. (2005) 147(2):192–6. 10.1016/J.JPEDS.2005.03.054 16126048

[39] Dani C Biadaioli R Bertini G Martelli E Rubaltelli FF. Probiotics feeding in prevention of urinary tract infection, bacterial sepsis and necrotizing enterocolitis in preterm infants. A prospective double-blind study. Biol Neonate. (2002) 82(2):103–8. 10.1159/000063096 12169832

[40] Manzoni P Mostert H Leonessa ML Priolo C Farina D Monetti C Oral supplementation with Lactobacillus casei subspecies rhamnosus prevents enteric colonization by Candida species in preterm neonates: a randomized study. Clin Infect Dis. (2006) 42(12):1735–42. 10.1086/504324 16705580

[41] Rojas MA Lozano JM Rojas MX Rodriguez VA Rondon MA Bastidas JA Prophylactic probiotics to prevent death and nosocomial infection in preterm infants. Pediatrics. (2012) 130(5):1113–20. 10.1542/PEDS.2011-3584 23071204

[42] Oncel MY Sari FN Arayici S Guzoglu N Erdeve O Uras N Lactobacillus reuteri for the prevention of necrotising enterocolitis in very low birthweight infants: a randomised controlled trial. Arch Dis Child Fetal Neonatal Ed. (2014) 99(2):110–5. 10.1136/ARCHDISCHILD-2013-304745 24309022

[43] Sari FN Dizdar EA Oguz S Erdeve O Uras N Dilmen U. Oral probiotics: Lactobacillus sporogenes for prevention of necrotizing enterocolitis in very low-birth weight infants: a randomized, controlled trial. Eur J Clin Nutr. (2011) 65(4):434–9. 10.1038/EJCN.2010.278 21245887

[44] Mihatsch WA Vossbeck S Eikmanns B Hoegel J Pohlandt F. Effect of Bifidobacterium lactis on the incidence of nosocomial infections in very-low-birth-weight infants: a randomized controlled trial. Neonatology. (2010) 98(2):156–63. 10.1159/000280291 20234140

[45] Costeloe K Hardy P Juszczak E Wilks M Millar MR. Bifidobacterium breve BBG-001 in very preterm infants: a randomised controlled phase 3 trial. Lancet. (2016) 387(10019):649–60. 10.1016/S0140-6736(15)01027-2 26628328

[46] Totsu S Yamasaki C Terahara M Uchiyama A Kusuda S. Bifidobacterium and enteral feeding in preterm infants: cluster-randomized trial. Pediatr Int. (2014) 56(5):714–9. 10.1111/PED.12330 24617812 PMC4285294

[47] Demirel G Erdeve O Celik IH Dilmen U. Saccharomyces boulardii for prevention of necrotizing enterocolitis in preterm infants: a randomized, controlled study. Acta Paediatr. (2013) 102(12):560–5. 10.1111/APA.12416 24028629

[48] Serce O Benzer D Gursoy T Karatekin G Ovali F. Efficacy of saccharomyces boulardii on necrotizing enterocolitis or sepsis in very low birth weight infants: a randomised controlled trial. Early Hum Dev. (2013) 89(12):1033–6. 10.1016/J.EARLHUMDEV.2013.08.013 24041815

[49] Jacobs SE Tobin JM Opie GF Donath S Tabrizi SN Pirotta M Probiotic effects on late-onset sepsis in very preterm infants: a randomized controlled trial. Pediatrics. (2013) 132(6):1055–62. 10.1542/PEDS.2013-1339 24249817

[50] Lin HC Hsu CH Chen HL Chung MY Hsu JF Lien RI Oral probiotics prevent necrotizing enterocolitis in very low birth weight preterm infants: a multicenter, randomized, controlled trial. Pediatrics. (2008) 122(4):693–700. 10.1542/PEDS.2007-3007 18829790

[51] Saengtawesin V Tangpolkaiwalsak R Kanjanapattankul W. Effect of oral probiotics supplementation in the prevention of necrotizing enterocolitis among very low birth weight preterm infants. J Med Assoc Thai. (2014) 97(Suppl 6):S20–5. PMID: 25391168

[52] Sowden M van Weissenbruch MM Bulabula ANH van Wyk L Twisk J van Niekerk E. Effect of a multi-strain probiotic on the incidence and severity of necrotizing enterocolitis and feeding intolerances in preterm neonates. Nutrients. (2022) 14(16):1–10. 10.3390/NU14163305 PMC9415863 36014810

[53] Rougé C Piloquet H Butel MJ Berger B Rochat F Ferraris L Oral supplementation with probiotics in very-low-birth-weight preterm infants: a randomized, double-blind, placebo-controlled trial. Am J Clin Nutr. (2009) 89(6):1828–35. 10.3945/AJCN.2008.26919 19369375

[54] Al-Hosni M Duenas M Hawk M Stewart LA Borghese RA Cahoon M Probiotics-supplemented feeding in extremely low-birth-weight infants. J Perinatol. (2012) 32(4):253–9. 10.1038/JP.2011.51 21546942

[55] Fernández-Carrocera LA Solis-Herrera A Cabanillas-Ayón M Gallardo-Sarmiento RB García-Pérez CS Montaño-Rodríguez R Double-blind, randomised clinical assay to evaluate the efficacy of probiotics in preterm newborns weighing less than 1500g in the prevention of necrotising enterocolitis. Arch Dis Child Fetal Neonatal Ed. (2013) 98(1):5–9. 10.1136/ARCHDISCHILD-2011-300435 22556209

[56] Kanic Z Micetic Turk D Burja S Kanic V Dinevski D. Influence of a combination of probiotics on bacterial infections in very low birthweight newborns. Wien Klin Wochenschr. (2015) 127(5):210–5. 10.1007/S00508-015-0845-0 26373743

[57] Shashidhar A Suman Rao PN Nesargi S Bhat S Chandrakala BS. Probiotics for promoting feed tolerance in very low birth weight neonates: a randomized controlled trial. Indian Pediatr. (2017) 54(5):363–7. 10.1007/S13312-017-1106-2 28368269

[58] Braga TD da Silva GAP de Lira PIC de Carvalho Lima M. Efficacy of Bifidobacterium breve and Lactobacillus casei oral supplementation on necrotizing enterocolitis in very-low-birth-weight preterm infants: a double-blind, randomized, controlled trial. Am J Clin Nutr. (2011) 93(1):81–6. 10.3945/AJCN.2010.29799 20980486

[59] Lin HC Su BH Chen AC Lin TW Tsai CH Yeh TF Oral probiotics reduce the incidence and severity of necrotizing enterocolitis in very low birth weight infants. Pediatrics. (2005) 115(1):1–4. 10.1542/PEDS.2004-1463 15629973

[60] Repa A Thanhaeuser M Endress D Weber M Kreissl A Binder C Probiotics (Lactobacillus acidophilus and Bifidobacterium bifidum) prevent NEC in VLBW infants fed breast milk but not formula [corrected]. Pediatr Res (2015) 77:381–88. 10.1038/PR.2014.192 25423074

[61] Martí M Spreckels JE Ranasinghe PD Wejryd E Marchini G Sverremark-Ekström E Effects of Lactobacillus reuteri supplementation on the gut microbiota in extremely preterm infants in a randomized placebo-controlled trial. Cell Rep Med. (2021) 2(3):1–11. 10.1016/J.XCRM.2021.100206 PMC7974321 33763652

[62] Denkel LA Schwab F Garten L Geffers C Gastmeier P Piening B. Protective effect of dual-strain probiotics in preterm infants: a multi-center time series analysis. PLoS One. (2016) 11(6):1–20. 10.1371/JOURNAL.PONE.0158136 PMC4917100 27332554

[63] Gómez-Rodríguez G Amador-Licona N Daza-Benítez L Barbosa-Sabanero G Carballo-Magdaleno D Aguilar-Padilla R Single strain versus multispecies probiotic on necrotizing enterocolitis and faecal IgA levels in very low birth weight preterm neonates: a randomized clinical trial. Pediatr Neonatol. (2019) 60(5):564–9. 10.1016/J.PEDNEO.2019.02.005 30898471

[64] Deshpande G Rao S Patole S. Probiotics for prevention of necrotising enterocolitis in preterm neonates with very low birthweight: a systematic review of randomised controlled trials. Lancet. (2007) 369(9573):1614–20. 10.1016/S0140-6736(07)60748-X 17499603

[65] Deshpande G Rao S Patole S Bulsara M. Updated meta-analysis of probiotics for preventing necrotizing enterocolitis in preterm neonates. Pediatrics. (2010) 125(5):921–30. 10.1542/PEDS.2009-1301 20403939

[66] Lau CSM Chamberlain RS. Probiotic administration can prevent necrotizing enterocolitis in preterm infants: a meta-analysis. J Pediatr Surg. (2015) 50(8):1405–12. 10.1016/J.JPEDSURG.2015.05.008 26216544

[67] Sharif S Meader N Oddie SJ Rojas-Reyes MX McGuire W. Probiotics to prevent necrotising enterocolitis in very preterm or very low birth weight infants. Cochrane Database Syst Rev. (2020) 10(10):1–133. 10.1002/14651858.CD005496.PUB5 PMC8094746 33058137

[68] Bi LW Yan BL Yang QY Li MM Cui HL. Probiotic strategies to prevent necrotizing enterocolitis in preterm infants: a meta-analysis. Pediatr Surg Int. (2019) 35(10):1143–62. 10.1007/S00383-019-04547-5 31420743

[69] Chang HY Chen JH Chang JH Lin HC Lin CY Peng CC. Multiple strains probiotics appear to be the most effective probiotics in the prevention of necrotizing enterocolitis and mortality: an updated meta-analysis. PLoS One. (2017) 12(2):1–14. 10.1371/JOURNAL.PONE.0171579 PMC5300201 28182644

[70] Fortmann I Marißen J Siller B Spiegler J Humberg A Hanke K Lactobacillus acidophilus/Bifidobacterium infantis probiotics are beneficial to extremely low gestational age infants fed human milk. Nutrients. (2020) 12(3):1–13. 10.3390/NU12030850 PMC7146289 32235769

[71] Samanta M Sarkar M Ghosh P Ghosh JK Sinha MK Chatterjee S. Prophylactic probiotics for prevention of necrotizing enterocolitis in very low birth weight newborns. J Trop Pediatr. (2009) 55(2):128–31. 10.1093/TROPEJ/FMN091 18842610

[72] O'Brien CE Meier AK Cernioglo K Mitchell RD Casaburi G Frese SA Early probiotic supplementation with B. infantis in breastfed infants leads to persistent colonization at 1 year. Pediatr Res. (2022) 91(3):627–36. 10.1038/S41390-020-01350-0 33762689 PMC8460680

[73] Gopalakrishna KP Macadangdang BR Rogers MB Tometich JT Firek BA Baker R Maternal IgA protects against the development of necrotizing enterocolitis in preterm infants. Nat Med. (2019) 25(7):1110–5. 10.1038/s41591-019-0480-9 31209335 PMC7424541

[74] Pammi M Suresh G. Enteral lactoferrin supplementation for prevention of sepsis and necrotizing enterocolitis in preterm infants. Cochrane Database Syst Rev. (2017) 6(6):1–49. 10.1002/14651858.CD007137.PUB5 PMC6481465 28658720

[75] Good M Sodhi CP Egan CE Afrazi A Jia H Yamaguchi Y Breast milk protects against the development of necrotizing enterocolitis through inhibition of toll-like receptor 4 in the intestinal epithelium via activation of the epidermal growth factor receptor. Mucosal Immunol. (2015) 8(5):1166–79. 10.1038/mi.2015.30 25899687 PMC4540669

[76] Probiotics & NEC: family-clinician communication is key - NEC society. Available at: https://necsociety.org/probiotics/ (Accessed November 12, 2022).

[77] Kunz AN Noel JM Fairchok MP. Two cases of Lactobacillus bacteremia during probiotic treatment of short gut syndrome. J Pediatr Gastroenterol Nutr. (2004) 38(4):457–8. 10.1097/00005176-200404000-00017 15085028

[78] Sadowska-Krawczenko I Paprzycka M Korbal P Wiatrzyk A Krysztopa-Grzybowska K Polak M Lactobacillus rhamnosus GG suspected infection in a newborn with intrauterine growth restriction. Benef Microbes. (2014) 5(4):397–402. 10.3920/BM2013.0074 25035097

[79] Zbinden A Zbinden R Berger C Arlettaz R. Case series of Bifidobacterium longum bacteremia in three preterm infants on probiotic therapy. Neonatology. (2015) 107(1):56–9. 10.1159/000367985 25402825

[80] Dani C Coviello C Corsini I Arena F Antonelli A Rossolini GM. Lactobacillus sepsis and probiotic therapy in newborns: two new cases and literature review. AJP Rep. (2016) 6(1):e25–9. 10.1055/S-0035-1566312 26929865 PMC4737628

[81] Chiang MC Chen CL Feng Y Chen CC Lien R Chiu CH. Lactobacillus rhamnosus sepsis associated with probiotic therapy in an extremely preterm infant: pathogenesis and a review for clinicians. J Microbiol Immunol Infect. (2021) 54(4):575–80. 10.1016/J.JMII.2020.03.029 32307246

[82] Bertelli C Pillonel T Torregrossa A Prod'hom G Julie Fischer C Greub G Bifidobacterium longum bacteremia in preterm infants receiving probiotics. Clin Infect Dis. (2015) 60(6):924–7. 10.1093/CID/CIU946 25472946

[83] Drago L Rodighiero V Celeste T Rovetto L de Vecchi E. Microbiological evaluation of commercial probiotic products available in the USA in 2009. J Chemother. (2010) 22(6):373–7. 10.1179/JOC.2010.22.6.373 21303743

[84] Toscano M de Vecchi E Rodighiero V Drago L. Microbiological and genetic identification of some probiotics proposed for medical use in 2011. J Chemother. (2013) 25(3):156–61. 10.1179/1973947812Y.0000000068 23783140

[85] Lewis ZT Shani G Masarweh CF Popovic M Frese SA Sela DA Validating bifidobacterial species and subspecies identity in commercial probiotic products. Pediatr Res. (2016) 79(3):445–52. 10.1038/PR.2015.244 26571226 PMC4916961

[86] Livia global announces voluntary recall of two lots of its liviaone liquid probiotics because of the potential for contamination with *Pseudomonas aeruginosa* | FDA. Available at: https://www.fda.gov/safety/recalls-market-withdrawals-safety-alerts/livia-global-announces-voluntary-recall-two-lots-its-liviaone-liquid-probiotics-because-potential (Accessed November 12, 2022).

[87] Out of an abundance of caution MaryRuth’s announces voluntary recall of two lots of its liquid probiotic for infants because of the potential for contamination with *Pseudomonas aeruginosa* | FDA. Available at: https://www.fda.gov/safety/recalls-market-withdrawals-safety-alerts/out-abundance-caution-maryruths-announces-voluntary-recall-two-lots-its-liquid-probiotic-infants (Accessed November 12, 2022).

[88] Probiotic formula recalled for potential salmonella contamination | Food Safety News. Available at: https://www.foodsafetynews.com/2012/09/probiotic-formula-recalled-for-potential-salmonella-contamination/ (Accessed November 12, 2022).

[89] Vallabhaneni S Walker TA Lockhart SR Ng D Diseases I Branch P Fatal gastrointestinal mucormycosis in a premature infant associated with a contaminated dietary supplement—connecticut, 2014. Morb Mortal Wkly Rep. (2015) 64(6):155. PMID: ; PMCID: 25695322 PMC4584706

[90] Ragan MV Wala SJ Goodman SD Bailey MT Besner GE. Next-generation probiotic therapy to protect the intestines from injury. Front Cell Infect Microbiol. (2022) 12:1–8. 10.3389/FCIMB.2022.863949 PMC9273849 35837474

[91] Orphan products: hope for people with rare diseases | FDA. Available at: https://www.fda.gov/drugs/information-consumers-and-patients-drugs/orphan-products-hope-people-rare-diseases (Accessed November 12, 2022). 14986582

[92] IBP-9414 for the prevention of necrotizing enterocolitis - the connection study - full text view - ClinicalTrials.gov. Available at: https://clinicaltrials.gov/ct2/show/NCT03978000 (Accessed December 7, 2022).

[93] IBT | FDA approves infant bacterial therapeutics’ request for a new orphan drug designation. Available at: https://ibtherapeutics.com/fda-approves-infant-bacterial-therapeutics-request-for-a-new-orphan-drug-designation/ (Accessed December 7, 2022).

[94] Neu J Moral TD Ferry J Guthrie S Nagy A Talati A Clinical outcomes correlating to a one-day shift in sustained feeding tolerance in very low birth weight infants in the ‘connection trial’. Br J Gastroenterol. (2022) 4(2):255–60. 10.31488/BJG.1000132

[95] Gibson GR Roberfroid MB. Dietary modulation of the human colonic microbiota: introducing the concept of prebiotics. J Nutr. (1995) 125(6):1401–12. 10.1093/JN/125.6.1401 7782892

[96] Gibson GR, Hutkins R, Sanders ME, Prescott SL, Reimer RA, Salminen SJ, et al. Expert consensus document: the international scientific association for probiotics and prebiotics (ISAPP) consensus statement on the definition and scope of prebiotics. Nat Rev Gastroenterol Hepatol. (2017) 14(8):491–502. 10.1038/NRGASTRO.2017.75 28611480

[97] Thomson P Medina DA Garrido D. Human milk oligosaccharides and infant gut bifidobacteria: molecular strategies for their utilization. Food Microbiol. (2018) 75:37–46. 10.1016/J.FM.2017.09.001 30056961

[98] Lewis ZT, Totten SM, Smilowitz JT, Popovic M, Parker E, Lemay DG, et al. Maternal fucosyltransferase 2 status affects the gut bifidobacterial communities of breastfed infants. Microbiome. (2015) 3(1). 10.1186/S40168-015-0071-Z PMC4412032 25922665

[99] Autran CA Schoterman MHC Jantscher-Krenn E Kamerling JP Bode L. Sialylated galacto-oligosaccharides and 2’-fucosyllactose reduce necrotising enterocolitis in neonatal rats. Br J Nutr. (2016) 116(2):294–9. 10.1017/S0007114516002038 27212112

[100] Kim WS Ohashi M Tanaka T Kumura H Kim GY Kwon IK Growth-promoting effects of lactoferrin on L. acidophilus and Bifidobacterium spp. Biometals. (2004) 17(3):279–83. 10.1023/B:BIOM.0000027705.57430.F1 15222478

[101] Pehlevan OS Benzer D Gursoy T Karatekin G Ovali F. Synbiotics use for preventing sepsis and necrotizing enterocolitis in very low birth weight neonates: a randomized controlled trial. Clin Exp Pediatr. (2020) 63(6):226–31. 10.3345/CEP.2019.00381 32023397 PMC7303425

[102] Manzoni P Meyer M Stolfi I Rinaldi M Cattani S Pugni L Bovine lactoferrin supplementation for prevention of necrotizing enterocolitis in very-low-birth-weight neonates: a randomized clinical trial. Early Hum Dev. (2014) 90(Suppl 1). 10.1016/S0378-3782(14)70020-9 24709463

[103] Güney-Varal İ Köksal N Özkan H Bağcı O Doğan P. The effect of early administration of combined multi-strain and multi-species probiotics on gastrointestinal morbidities and mortality in preterm infants: a randomized controlled trial in a tertiary care unit. Turk J Pediatr. (2017) 59(1):13–9. 10.24953/TURKJPED.2017.01.003 29168358

[104] Huoman J Martínez-Enguita D Olsson E Ernerudh J Nilsson L Duchén K Combined prenatal Lactobacillus reuteri and *ω*-3 supplementation synergistically modulates DNA methylation in neonatal T helper cells. Clin Epigenetics. (2021) 13(1):135. 10.1186/S13148-021-01115-4 34193262 PMC8247185

[105] Tsilingiri K Rescigno M. Postbiotics: what else? Benef Microbes. (2013) 4(1):101–7. 10.3920/BM2012.0046 23271068

[106] Żółkiewicz J Marzec A Ruszczyński M Feleszko W. Postbiotics-A step beyond Pre- and probiotics. Nutrients. (2020) 12(8):1–17. 10.3390/NU12082189 PMC7468815 32717965

[107] Meng D Sommella E Salviati E Campiglia P Ganguli K Djebali K Indole-3-lactic acid, a metabolite of tryptophan, secreted by Bifidobacterium longum subspecies infantis is anti-inflammatory in the immature intestine. Pediatr Res. (2020) 88(2):209–17. 10.1038/S41390-019-0740-X 31945773 PMC7363505

[108] Branda SS Vik Å Friedman L Kolter R. Biofilms: the matrix revisited. Trends Microbiol. (2005) 13(1):20–6. 10.1016/J.TIM.2004.11.006 15639628

[109] Salas-Jara MJ Ilabaca A Vega M García A. Biofilm forming Lactobacillus: new challenges for the development of probiotics. Microorganisms. (2016) 4(3):35. 10.3390/MICROORGANISMS4030035 27681929 PMC5039595

[110] Gao K Liu L Dou X Wang C Liu J Zhang W Doses Lactobacillus reuteri depend on adhesive ability to modulate the intestinal immune response and metabolism in mice challenged with lipopolysaccharide. Sci Rep. (2016) 6(1):1–12. 10.1038/srep28332 27323686 PMC4915000

[111] Olson JK Navarro JB Allen JM McCulloh CJ Mashburn-Warren L Wang Y Harvesting the benefits of biofilms: a novel probiotic delivery system for the prevention of necrotizing enterocolitis. J Pediatr Surg. (2016) 51(6):936–41. 10.1016/J.JPEDSURG.2016.02.062 27032609

[112] Al-Hadidi A Navarro J Goodman SD Bailey MT Besner GE. Lactobacillus reuteri in its biofilm state improves protection from experimental necrotizing enterocolitis. Nutrients. (2021) 13(3):1–12. 10.3390/nu13030918 PMC8000340 33809097

[113] Chua KJ Kwok WC Aggarwal N Sun T Chang MW. Designer probiotics for the prevention and treatment of human diseases. Curr Opin Chem Biol. (2017) 40:8–16. 10.1016/J.CBPA.2017.04.011 28478369

[114] Yang G Jiang Y Yang W Du F Yao Y Shi C Effective treatment of hypertension by recombinant Lactobacillus plantarum expressing angiotensin converting enzyme inhibitory peptide. Microb Cell Fact. (2015) 14(1):1–9. 10.1186/S12934-015-0394-2/TABLES/3 26691527 PMC4687296

[115] Wei P Yang Y Li T Ding Q Sun H. A engineered Bifidobacterium longum secreting a bioative penetratin-glucagon-like peptide 1 fusion protein enhances glucagon-like peptide 1 absorption in the intestine. J Microbiol Biotechnol. (2015). PMID: . [Epub ahead of print] 25674803

[116] Guo S Yan W McDonough SP Lin N Wu KJ He H The recombinant Lactococcus lactis oral vaccine induces protection against C. difficile spore challenge in a mouse model. Vaccine. (2015) 33(13):1586–95. 10.1016/J.VACCINE.2015.02.006 25698490

[117] Koh E Hwang IY Lee HL de Sotto R Lee JWJ Lee YS Engineering probiotics to inhibit clostridioides difficile infection by dynamic regulation of intestinal metabolism. Nat Commun. (2022) 13(1):1–13. 10.1038/s41467-022-31334-z 35787625 PMC9253155

[118] Paton AW Morona R Paton JC. Designer probiotics for prevention of enteric infections. Nat Rev Microbiol. (2006) 4(3):193–200. 10.1038/NRMICRO1349 16462752

[119] Mihi B Gong Q Nolan LS Gale SE Goree M Hu E Interleukin-22 signaling attenuates necrotizing enterocolitis by promoting epithelial cell regeneration. Cell Rep Med. (2021) 2(6):100320. 10.1016/J.XCRM.2021.100320 34195684 PMC8233697

[120] Espinal A Epperly MW Mukherjee A Fisher R Shields D Wang H Intestinal radiation protection and mitigation by second-generation probiotic Lactobacillus-reuteri engineered to deliver interleukin-22. Int J Mol Sci. (2022) 23(10). 10.3390/IJMS23105616 35628427 PMC9145862

